# Risk reduction behaviours following the detection of unexpected drugs through community drug checking: a cross-sectional study

**DOI:** 10.1186/s12954-025-01374-x

**Published:** 2026-03-05

**Authors:** Samuel Tobias, Cameron Grant, Mark Lysyshyn, Perrine Roux, Evan Wood, Thomas Kerr, Lianping Ti

**Affiliations:** 1 British Columbia Centre on Substance Use, 400–1190 Hornby Street, Vancouver, BC V6Z 2K5 Canada; 2 School of Population and Public Health, University of British Columbia, 2206 East Mall, Vancouver, BC V6T 1Z3 Canada; 3 Vancouver Coastal Health Authority, 520 West 6th Avenue, Vancouver, BC V5Y 1L3 Canada; 4 Sciences Économiques et Sociales de la Santé & Traitement de l’information Médicale, Inserm, 19-21 Boulevard Jean Moulin, 13005 Marseille, France; 5 Department of Medicine, University of British Columbia, 317-2194 Health Sciences Mall, Vancouver, BC V6T 1Z3 Canada

**Keywords:** Drug checking, Harm reduction, Illicit drugs, Drug adulteration, Behaviour change

## Abstract

**Background:**

Community-based drug checking services have scaled up in response to the unregulated drug toxicity crisis across North America. We sought to assess the relationship between the detection of unexpected active drugs in a drug checking sample and subsequent engagement in risk reduction practices.

**Methods:**

We used data from a cross-sectional study of people who used community-based drug checking services in British Columbia, Canada (March 2020–July 2024). We constructed multivariable logistic regression models to examine the relationship between the detection of unexpected active drugs (i.e., compounds that have psychoactive properties) in samples brought for analysis (using combination Fourier-transform infrared spectroscopy and immunoassay strips) and engagement in subsequent risk reduction practices (e.g., dose reduction, disposal of the drug).

**Results:**

In total, 447 individuals were included: 174 (38.9%) reported detection of unexpected active drugs in their drug checking sample with the most common being benzodiazepines in expected opioid samples. The most common risk reduction behaviours were to dispose of the drug without using it (*n* = 24) and taking a smaller dose (*n* = 11). Through multivariable logistic regression, we found a positive association between detection of unexpected active drugs and engagement in risk reduction practices (adjusted odds ratio = 2.24; 95% confidence interval: 1.30–3.87).

**Conclusion:**

Individuals who detected unexpected active drugs in their sample had higher odds of engaging in risk reduction practices. These findings highlight the potential of drug checking services as a harm reduction tool within a suite of services offered to combat the unpredictable and unregulated drug supply.

## Introduction

The unregulated drug supply in North America continues to evolve with the introduction of new psychoactive substances and potent fentanyl analogues [1, 2]. Risks associated with accessing unregulated drugs are not only limited to people who are using opioids; stimulants are increasingly being found to contain fentanyl analogues [3], posing particular risk for opioid-naïve people who use stimulants. In British Columbia (BC), the Canadian province with the highest unregulated drug-related mortality rate [4], fentanyl and its analogues continue to dominate the toxic drug supply, as both a desired substance and unexpected adulterant, contributing to increased non-fatal poisoning events and record numbers of preventable deaths [5, 6]. This unprecedented crisis has spurred the expansion of harm reduction initiatives, including supervised consumption sites, expanded access to opioid agonist therapies, and drug checking services, in an attempt to mitigate the devastating toll of unregulated drug toxicity (overdose) [7]. 

Unlike their pharmaceutical counterparts, unregulated drugs contain unknown ingredients in variable, unreliable amounts or concentrations. For example, fentanyl concentrations in the unregulated opioid supply have been shown to range from trace levels to over 70%, with significant batch-to-batch variability, even within the same geographic region [8]. Compared to regulated pharmaceuticals subject to strict production oversight, unregulated drugs are often produced in clandestine laboratories and then prepared for distribution in small-scale, decentralized settings. In fact, a recent study from Vancouver, BC found that nearly one in five people who use drugs reported engaging in drug selling [9]. The persistent unpredictability of drug contents results in people relying heavily on trust in their sellers and word of mouth [10], when in fact, having a regular source does not necessarily result in more consistent drugs [11]. 

In an otherwise opaque market, drug checking services offer individuals and their networks some level of consumer protection by informing them about the contents of their analyzed drug sample [12]. Drug checking services, which help counter the effects of the unregulated drug toxicity crisis, have been offered in BC since 2017 and have become one of the province’s key responses in the ongoing public health response [13]. They allow drug sellers to understand the composition of the drugs they are selling [14], and people who use drugs themselves to make decisions about their substance use with the overarching goal of promoting risk-reducing behaviour changes. Previous studies have explored how individuals who access drug checking services respond to results of analysis (e.g., change their intention or seek more information) [12]. However, much of this research relies on hypothetical situations or took place in music festival settings where recreational drug use most often takes place [15, 16]. As such, there remains a gap in in our understanding both of: (1) how people in community settings affected by the toxic drug supply respond to drug checking results, and (2) how common are risk reducing behaviours taking place following receipt of drug checking results.

The objective of this study is to assess whether the detection of unexpected active drugs through community drug checking is associated with subsequent engagement in risk reduction behaviours among people who use the service. By examining behavioural responses to unexpected results, this research aims to enhance our understanding of how drug checking services may effectively improve safety and mitigate risks among people who use drugs. These findings may help inform the development and refinement of harm reduction strategies to optimally address the challenges posed by the evolving unregulated drug supply.

## Methods

### Study design

Data were derived from a cross-sectional study that was undertaken across 22 community harm reduction sites offering drug checking services in BC. The majority of sites offered comprehensive harm reduction services (e.g., syringe service programs, supervised consumption) and a minority were standalone drug checking sites. Drug checks across all sites were primarily conducted using a combination of Fourier-transform infrared spectroscopy and immunoassay strips (fentanyl and benzodiazepines). Individuals were eligible to participate in the survey if they met the following criteria: (1) 14 years of age or older; (2) accessed a drug checking service in the last six months; (3) could communicate in English; and (4) provided informed consent. Recruitment was through convenience sampling using word-of mouth from study participants and invitations posed by drug checking technicians, as well as from printed communication products (e.g., business cards) distributed at the harm reduction sites. We conducted the surveys both over the phone and in-person, depending on the location. The questionnaire was administered by research assistants and interviewers, some of whom were people with lived or living experience of substance use and drug checking service use. Interviewers were never the same person as the drug checking technician. The questionnaire elicited information on socio-demographics, substance use patterns, drug checking experience, experiences with negative reactions to drug use, and health and harm reduction service utilization. Questions with respect to risk reduction practices were specific to the drug most recently checked by the participant. Participants were provided a CAD$25 cash honorarium for completing the study.

### Study sample

We included all individuals who completed the questionnaire and therefore recently used a drug checking service. To measure enacted behaviour change, we removed participants who reported that they had not yet used the checked drug at the time of the interview (2.4%), except for those that opted to not use it. Interviews took place between March 2020 and July 2024.

### Variable selection

The primary outcome variable was engagement in a risk reduction practice following drug checking service utilization (yes vs. no), dichotomized from the question, “What did you do with your drugs when you got your result?” Practices we defined as risk reducing included: took a tester shot, took less, buffed (diluted) their drug, did not use alone, split into smaller doses, disposed of, used over a longer period of time, used a supervised consumption site or overdose prevention site, changed method of use, gave it away, sold the drug, or self-reported ‘others’ that met our subjective threshold of risk reduction (e.g., “Got a nurse to help,” or “Did a smoke test”). Responses were collected as select all that apply. The list of behaviours was developed in consultation with peer research associates, and the survey was piloted with a small number of participants who provided feedback. As a sensitivity analysis, we recategorized responses related to ‘gave it away’ and ‘sold’ into the ‘no’ category given that while these may be a way to reduce risk among the participant themselves, it may have increased the risk of a negative health harm for someone else.

The main exposure of interest was the detection of at least one unexpected active drug in the drug checking sample (yes vs. no). This variable was generated reflecting the presence of a discrepancy between the questions, “What drug did you check today (or the last time) you checked your drugs?” and, “What was the result?” We assessed whether the drug checking results included any active drugs that were not what the participant expected when they brought the drug in for checking. To define unexpected active drugs, we intentionally only included compounds that have psychoactive properties (e.g., fentanyl, benzodiazepine, etizolam) or sensation-inducing properties (e.g., lidocaine, benzocaine) as we hypothesized that drug use would not likely change following the detection of compounds without these properties (e.g., mannitol, dimethylsulfone). We considered caffeine non-psychoactive as it is a very common drug additive for opioids in our study’s setting [17]. Responses were coded as ‘yes’ if there were active drugs present that were not the drug they expected, regardless of whether the drug they expected was present. For example, if a participant indicated that they expected fentanyl and the results showed a positive detection of fentanyl and benzodiazepine, then it was considered as having an unexpected active drug (i.e., benzodiazepine). Similarly, if the participant indicated that they expected cocaine and the results showed a positive detection of methamphetamine, it would also be considered as having an unexpected active drug (i.e., methamphetamine).

Secondary variables used to describe the study participants and various confounding factors that might have an effect on the relationship between the detection of an unexpected active drug and engagement in a risk reduction behaviour following drug checking results were considered. These included: age (per year older), gender (women vs. gender minority vs. men [reference]), ethnicity or ancestry (white vs. Indigenous, Black, and Persons of Colour), income assistance (yes vs. no), frequency of unregulated opioid use ( > = weekly vs. < weekly vs. no use [reference]), frequency of stimulant use ( > = weekly vs. < weekly vs. no use [reference], have a regular source of drugs (yes vs. no), drug checked was an opioid (yes vs. no), and drug checked was a stimulant (yes vs. no).

### Statistical analyses

First, we descriptively examined our primary (i.e., detection of an unexpected active drug) and secondary variables, stratified by engagement in a risk reduction practice. Then, we conducted bivariable and multivariable logistic regression models to estimate the relationship between detection of an unexpected active drug and engagement in a risk reduction practice. We performed two sensitivity analyses: we re-ran the multivariable model with our alternate outcome variable definition which removed ‘gave it away’ and ‘sold’ as a risk reduction practice. Then, given that benzodiazepine-adulteration of unregulated opioids is common in BC and our concern these samples may be dominating our results, we excluded participants whose drug sample contained unexpected benzodiazepines. In a sub-analysis, we explored among those who had an unexpected active drug in their sample, what they expected the drug to be compared to what their result was. All statistical analyses were conducted using SAS (Version 9.4; SAS Institute Inc., Cary, NC).

## Results

### Study sample characteristics

In total, 447 individuals were included in the analysis. The median participant age was 43 years (interquartile range of 16); 32.5% of participants were women and 2.0% identified as a gender minority. Among all participants, 121 of 447 (27.1%) identified as Indigenous, Black, or a Person of Colour. As broader drug checking services are anonymous in BC, we are unable to comment on if this sample is consistent with the universe of people who access drug checking across the province. We excluded participants who reported not having made a decision to use their drug yet (e.g., “Haven’t used them since getting them checked”) (*n* = 11) to capture enacted behaviours relating to checked drugs. Table 1 reports characteristics of included participants. Among included participants, 174 (38.9%) reported the detection of unexpected active drugs in their drug checking sample; this proportion was highest in the first calendar year of the study (2021, 49.1%) and lowered to a consistent level in subsequent years (2022, 37.5%; 2023, 37.0%; 2024, 38.0%). Of these, 42 (24.1%) reported engaging in a risk reduction practice after their drug check, compared to 13.5% of those without unexpected drugs. The most common risk reduction behaviour was to dispose of the drug without using it (*n* = 24), followed by taking a smaller dose (*n* = 11), giving it away (*n* = 11), changing method of use (*n* = 4), or not using alone (*n* = 4).

**Table 1:** Participant characteristics, stratified by engagement in a risk reduction practice following receipt of drug checking results (*n* = 447)

Characteristic	Total (%) (*n* = 447)	Risk reduction practice		
		Yes (%) (*n* = 79)	No (%) (*n* = 368)	*p*-value
Main exposure variable				
Unexpected active drugs	174 (38.9)	42 (9.4)	132 (29.5)	0.02
Secondary variables				
Age (median, quartile 1–3)	43 (36–52)	43 (36–52)	43 (36–52)	0.98
Gender				0.58
Man	292 (65.5)	54 (12.1)	238 (53.4)	
Woman	145 (32.5)	25 (5.6)	120 (26.9)	
Gender minority	9 (2.0)	0 (0.0)	9 (2.0)	
Ethnicity/ancestry				0.37
Black, Indigenous, Person of Colour	121 (27.1)	17 (3.8)	104 (23.3)	
White	326 (72.9)	62 (13.9)	264 (59.1)	
Income assistance	211 (47.2)	33 (7.4)	178 (39.8)	0.44
≥ Weekly unregulated opioid use	351 (78.5)	65 (14.5)	286 (64.0)	0.81
≥ Weekly stimulant use	364 (81.4)	64 (14.3)	300 (67.1)	0.65
Have a regular source of drugs	359 (81.8)	67 (15.3)	292 (66.5)	0.23
Drug checked an opioid	310 (69.4)	53 (11.9)	257 (57.5)	0.79
Drug checked a stimulant	140 (31.3)	25 (5.6)	115 (25.7)	1.00

*p*-values calculated using t-test, chi-square test, or Fisher’s exact test where appropriate

**Table 2:** Logistic regression models estimating the relationship between detection of an unexpected active drug and engagement in a risk reduction practice following receipt of drug checking results (*n* = 438

	Adjusted model		Sensitivity analysis model*	
	Adjusted odds ratio (95% confidence interval)	*p*-value	Adjusted odds ratio (95% confidence interval)	*p*-value
Primary exposure variable				
Unexpected active drug				
(yes vs. no)	2.24 (1.30–3.87)	0.004	2.59 (1.35–4.97)	0.004
Additional covariates in adjusted models				
Age				
Years	1.00 (0.98–1.02)	0.987	1.01 (0.98–1.04)	0.580
Gender				
Man	Ref.		Ref.	
Woman	0.86 (0.49–1.48)	0.577	1.00 (0.52–1.91)	0.997
Gender minority	Inf.	0.958	Inf.	
Unregulated opioid use				
Never	Ref.		Ref.	
Less than weekly	3.02 (0.60–15.31)	0.181	1.26 (0.21–7.46)	0.800
Greater than weekly	3.38 (0.71–15.94)	0.125	2.69 (0.55–13.14)	0.221
Unregulated stimulant use				
Never	Ref.		Ref.	
Less than weekly	Inf.	0.958	Inf.	0.963
Greater than weekly	Inf.	0.959	Inf.	0.963
Regular source of drugs				
No	Ref.		Ref.	
Yes	1.93 (0.90–4.14)	0.090	1.20 (0.53–2.74)	0.662
Drug checked an opioid				
No	Ref.		Ref.	
Yes	0.55 (0.26–1.18)	0.125	0.34 (0.14–0.80)	0.014
Drug checked a stimulant				
No	Ref.		Ref.	
Yes	0.73 (0.35–1.53)	0.407	0.65 (0.27–1.55)	0.334

*Sensitivity analysis refers to the recategorization of the main outcome where ‘gave it away’ and ‘sold to’ were categorized as not a risk reduction practice

Covariates with infinite adjusted odds ratios reflect perfect prediction of the outcome due to small cell counts

### Risk-reducing behaviours following the detection of unexpected drugs

Through multivariable logistic regression, we found a positive association between detection of unexpected active drugs and the engagement in a risk reduction practice (adjusted odds ratio = 2.24; 95% confidence interval: 1.30–3.87) compared to those without unexpected active drugs (Table 2). The unadjusted odds ratio was 2.03 (95% confidence interval: 1.24–3.31), indicating that the association persisted after adjustment for covariates. The sensitivity analysis with an alternate definition of risk reducing behaviour indicated robustness of results (adjusted odds ratio = 2.59; 95% confidence interval: 1.35–4.97). Following the removal of participants who reported unexpected benzodiazepines, the results remained consistent (adjusted odds ratio = 2.31; 95% confidence interval: 1.09–4.88).

### Unexpected drugs detected

Reported drug checking results with unexpected findings spanned opioid, stimulant, and depressant categories. Among samples expected to contain fentanyl (or “down,” as it is colloquially known in BC) and no other active drugs, reported unexpected drugs included benzodiazepines (35.2%), methamphetamine (2.4%), and other pharmaceutical opioids (1.0%). Among expected methamphetamine samples, unexpected results included fentanyl (4.5%), crack cocaine (3.3%), and benzodiazepines (1.5%). For cocaine and crack cocaine, unexpected results included benzodiazepines (8.1%) and benzocaine (2.7%). Among all participants, 54 (12.1%) people’s drugs did not contain their expected drug in any amount, and 34 (7.6%) had an unexpected active drug in the absence of their expected drug (i.e., replacement as opposed to adulteration).

## Discussion

In a survey of people in BC who accessed drug checking services, 24.1% of those who received unexpected findings reported engaging in risk reduction behaviours, corresponding to 124% higher odds compared to those without unexpected active drugs. These results describe the prevalence of unexpected drugs in this setting and highlight the potential role of receiving unexpected results in influencing the uptake of risk reduction practices.

This research contributes novel findings to the evidence base examining drug checking as a harm reduction strategy in the context of a toxic, unregulated drug supply. Among our study’s participants, 38.9% reported the detection of an unexpected active drug in submitted sample, with the majority being benzodiazepines in purported opioids samples. The decrease in unsuspected drug reports from 2022 to 2023 is consistent with the growing trend of benzodiazepine-adulteration of opioid during this period and the stabilization or saturation in subsequent years [18]. Given the well-established risk of toxicity associated with benzodiazepine-opioid combinations [2], these findings emphasize the importance of understanding how people respond to drug checking results in real-world settings. In these situations, drug checking technicians typically provide harm reduction counselling consistent with local health authorities [19], such as advising participants to use smaller test doses, avoid using alone, ensure naloxone is available, and be aware of the heightened sedation risk when combining depressants.

Much of the evidence informing drug checking service delivery and funding has come from qualitative research on willingness among potential service users [20, 21], has been based on intentions following receipt of results [22–24], or intention following a hypothetical situation [25, 26]. Research that has been conducted regarding enacted, as opposed to intended, behaviours has been largely limited to recreational drug settings (e.g., “party drugs” such as ecstasy) [15, 16], or been based on the binary results of a fentanyl test strip [27]. Indeed, a 2022 systematic review of research on drug checking services indicated of the 90 publications about drug checking, 22 (24.4%) reported on intended behavioural change, while 16 (17.8%) were based on enacted (actual) responses to receiving drug checking results; however, none of these 16 studies were conducted in community settings with a more sophisticated technology than fentanyl test strips [12]. Our study contributes to this literature by underscoring the value of spectroscopic drug checking technologies in identifying active drug components beyond qualitative test strips and their potential role in shaping subsequent behaviour change, particularly when service models preclude the use of a more sophisticated technology such as mass spectrometry.

A further strength of our study is the explicit differentiation between unexpected active and inactive drugs. Previous research has often treated unexpected findings as a broad category without distinguishing whether the compounds detected had psychoactive properties [22, 23]. This distinction is critical, as unexpected active drugs may pose greater immediate risks (e.g., adverse drug reactions or interactions) and are more likely to influence behavioural change compared to inactive adulterants or inert agents. By incorporating this level of detail, our study provides a more precise understanding of how different types of unexpected findings may influence harm reduction behaviours among those who test their drugs, contributing an important perspective to the literature on drug checking.

The behavioural responses observed in this study may be understood through the health belief model, a framework for explaining health-related decision-making [28]. According to the model, individuals are more likely to adopt protective behaviours when they perceive themselves to be personally susceptible to harm, believe the consequences of that harm are serious, and see clear benefits to taking action that outweigh potential barriers [29]. In the context of this study, receiving an unexpected drug checking result, such as the detection of benzodiazepines in presumed opioid samples, may increase perceived susceptibility to overdose and the perceived severity of that risk. These results may act as a cue to action, prompting individuals to engage in risk reduction behaviours such as discarding the drug, using less, or seeking supervised consumption. This judgement of severity may explain how most participants with unexpected drugs reported no behaviour change; our study recruited from community sites with roughly half of participants reporting income assistance and therefore disposing of drugs may be seen as a costly endeavour. It may also explain the pairing of harm reduction practices, such as the participants who reported not finding unexpected drugs but still reported a risk reduction behaviour, as the act of drug checking has been shown to be associated with additional harm reducing actions [25]. The process of submitting a sample, receiving information about its composition, and engaging with harm reduction staff may prompt reflection on safety and promote behavioural change, even when results are reassuring. While this study did not explicitly measure these constructs, the findings are consistent with the model’s emphasis on perceived threat, benefits, barriers, and cues as central drivers of behaviour change.

Our study has limitations that must be considered. First, sampling was based on volunteer participants who self-recruited following a drug check and therefore may not be generalizable to the broader population of people who use drugs in BC or other settings. Indeed, previous studies in BC have estimated that 21–28% of persons who use drugs in the province avail themselves of drug checking services [9, 25]. In this context, future research should examine the impact on drug markets and on drug sellers accessing drug checking services, as this may have the potential to influence the composition of the unregulated supply more broadly. Even for people who do not personally use drug checking services, changes in drug composition or distribution practices by sellers may indirectly shape the drug supply and associated risks at the population level. Secondly, although some participants completed the survey shortly after their drug check, some may have conducted it sometime later (but within six months), resulting in recall bias. There further exists the possibility of social desirability bias skewing the findings, though we know of no reason why having unexpected findings would lead to non-causal differential reporting. Additionally, behavioural responses to unexpected results likely vary according to the class of adulterant identified; for example, a stimulant unexpectedly containing fentanyl may elicit a different response than an opioid containing another depressant, but our study was not powered to assess such class-specific effects. Finally, although we report covariate effect estimates in Table 2 for transparency, certain covariate categories (e.g., gender minority participants; *n* = 9) were small. Sparse data in these categories may affect model stability and contribute slightly to imprecision in adjusted estimates, even when such covariates are included only for adjustment rather than effect estimation. However, the adjusted odds ratios were consistent with both unadjusted estimates and the sensitivity analysis.

Taken together, our findings suggest that unexpected active drugs present across a diversity of submitted drug types, including the detection of unexpected benzodiazepines in opioid samples, were associated with significantly higher odds of reported risk reduction behaviours. Given the established risks of unexpected opioids adulterating stimulants and benzodiazepines in combination with opioids, this behavioural response is encouraging and underscores the potential for drug checking to inform safer use practices. These findings reinforce the value of drug checking technologies capable of detecting a wide spectrum of active components and suggest opportunities for more tailored harm reduction messaging. However, given the limitations of voluntary participation and self-reported data, further research is needed to understand how drug checking influences decision-making across diverse user groups and contexts. Future studies should also consider how different types of unexpected results are interpreted and acted upon by people who use drugs, suppliers, and service providers alike.

## Data Availability

The data that support the findings of this study are not available due to ethical considerations as the participants did not consent to public data sharing.
